# Risco de violência em pessoas idosas: o papel das interações sociais, do apoio social e da qualidade de vida no estudo longitudinal EpiFloripa Idoso

**DOI:** 10.1590/0102-311XPT201825

**Published:** 2026-05-18

**Authors:** Adriana Lopes Elias, Anna Quialheiro Abreu da Silva, Eliana Funk, Larissa Pruner Marques, Thamara Hubler Figueiró, Danúbia Hillesheim, Sheila Rubia Lindner, Eleonora d’Orsi

**Affiliations:** 1 Universidade Federal de Santa Catarina, Florianópolis, Brasil.; 2 Instituto Politécnico de Saúde do Norte, Braga, Portugal.; 3 Programa de Computação Científica, Fundação Oswaldo Cruz, Rio de Janeiro, Brasil.

**Keywords:** Idoso, Apoio Social, Qualidade de Vida, Violência contra Pessoa Idosa, Aged, Social Support, Quality of Life, Senior Neglect, Anciano, Apoyo Social, Calidad de Vida, Maltrato de Anciano

## Abstract

O objetivo deste estudo foi estimar a associação entre o risco de violência e dimensões relacionadas às interações sociais, ao apoio social e à qualidade de vida entre os participantes do estudo longitudinal EpiFloripa Idoso. Foram analisadas entrevistas das ondas 1 (2009/2010; n = 1.702), 2 (2013/2014; n = 1.197) e 3 (2017/2019; n = 1.335) da coorte de pessoas idosas (≥ 60 anos) residentes na área urbana de Florianópolis, Santa Catarina, Brasil. O risco de violência doméstica também caracterizado pela condição de vulnerabilidade à violência, desfecho deste estudo, foi avaliado pela escala *Hwalek-Sengstock Elder Abuse Screening Test*, associado ao uso de internet, trabalho remunerado, participação em grupos sociais, apoio social (*Escala de Apoio Social do Medical Outcomes Study*) e qualidade de vida (CASP-19 - *Control, Autonomy, Self-realization and Pleasure*). As associações foram estimadas por um modelo de análise longitudinal, equações de estimação generalizadas, ajustadas por sexo, idade, escolaridade e renda. A prevalência do desfecho foi de 31,4% (IC95%: 27,8; 35,2) na onda 2 e 22,3% (IC95%: 19,1; 25,9) na onda 3. Observou-se menor ocorrência do desfecho entre participantes com trabalho remunerado (OR = 0,67; IC95%: 0,49; 0,91), participação em grupos sociais (OR = 0,76; IC95%: 0,63; 0,92) e apoio social elevado (OR = 0,44; IC95%: 0,33; 0,59). Níveis superiores de qualidade de vida apresentaram menor vulnerabilidade à violência, tanto em controle/autonomia (OR = 0,30; IC95%: 0,23; 0,38) quanto em autorrealização/prazer (OR = 0,44; IC95%: 0,34; 0,55). Maiores interações sociais, apoio social e elevados níveis de qualidade de vida associaram-se a menor ocorrência de violência, contribuindo para a identificação de fatores protetores relevantes no envelhecimento.

## Abstract (en)

This study aimed to estimate the association between the risk of violence and dimensions related to social interactions, social support, and quality of life among participants in the EpiFloripa Aging longitudinal study. Interviews from the first (2009/2010; n = 1,702), second (2013/2014; n = 1,197), and third waves (2017/2019; n = 1,335) of the cohort of older adults (≥ 60 years) living in the urban area of Florianópolis, Santa Catarina State, Brazil, were analyzed. The risk of domestic violence, also characterized by the condition of vulnerability to violence, the outcome of this study, was assessed by the scale *Hwalek-Sengstock Elder Abuse Screening Test*, associated with the use of the internet, paid work, participation in social groups, social support (*Social Support Scale of the Medical Outcomes Study*) and quality of life (CASP-19 − *Control, Autonomy, Self-realization and Pleasure*). Associations were estimated using a longitudinal analysis model, Generalized Estimation Equations, adjusted for sex, age, education, and income. The prevalence of the outcome was 31.4% (95%CI: 27.8; 35.2) in the second wave and 22.3% (95%CI: 19.1; 25.9) in the third wave. There was a lower occurrence of violence among participants with paid work (OR = 0.67; 95%CI: 0.49; 0.91), participation in social groups (OR = 0.76; 95%CI: 0.63; 0.92) and high social support (OR = 0.44; 95%CI: 0.33; 0.59). Individuals with better quality of life showed lower vulnerability to violence, both in control/autonomy (OR = 0.30; 95%CI: 0.23; 0.38) and self-realization/pleasure (OR = 0.44; 95%CI: 0.34; 0.55). Greater social interactions, social support and better quality of life were associated with a lower occurrence of violence, contributing to the identification of relevant protective factors in aging.

## Resumen

El objetivo de este estudio fue estimar la asociación entre el riesgo de violencia y las dimensiones relacionadas con las interacciones sociales, el apoyo social y la calidad de vida entre los participantes del estudio longitudinal EpiFloripa Idoso. Se analizaron entrevistas de las olas 1 (2009/2010; n = 1.702), 2 (2013/2014; n = 1.197) y 3 (2017/2019; n = 1.335) de la cohorte de adultos mayores (≥ 60 años) residentes en el área urbana de Florianópolis, Santa Catarina, Brasil. El riesgo de violencia doméstica caracterizado también por la condición de vulnerabilidad a la violencia, desenlace de este estudio, se evaluó según la escala *Hwalek-Sengstock Elder Abuse Screening Test*, asociado al uso de internet, trabajo remunerado, participación en grupos sociales, apoyo social (*Escala de Apoyo Social, Medical Outcomes Study*) y calidad de vida (CASP-19 − *Control, Autonomy, Self-realization and Pleasure*). Las asociaciones se estimaron utilizando un modelo de análisis longitudinal, ecuaciones de estimación generalizadas, ajustadas por sexo, edad, educación e ingresos. La prevalencia del desenlace fue del 31,4% (IC95%: 27,8; 35,2) y en la ola 2 fue del 22,3% (IC95%: 19,1; 25,9) en la ola 3. Se observó una menor incidencia del desenlace entre los participantes con trabajo remunerado (OR = 0,67; IC95%: 0,49; 0,91), participación en grupos sociales (OR = 0,76; IC95%: 0,63; 0,92) y alto apoyo social (OR = 0,44; IC95%: 0,33; 0,59). Los niveles más altos de calidad de vida se asociaron con una menor vulnerabilidad a la violencia, tanto en términos de control/autonomía (OR = 0,30; IC95%: 0,23; 0,38) así como en la autorrealización/placer (OR = 0,44; IC95%: 0,34; 0,55). Una mayor interacción social, apoyo social y niveles más altos de calidad de vida se asociaron con una menor incidencia de violencia, lo que contribuyó a la identificación de factores protectores relevantes en el envejecimiento.

## Introdução

O envelhecimento populacional é um fenômeno recente em termos históricos e impõe desafios crescentes aos sistemas de saúde e proteção social ^1^. Estimativas globais indicam que, até 2030, o número de pessoas idosas alcançará 1,4 bilhão, podendo dobrar até 2050, quando uma em cada cinco pessoas terá 60 anos ou mais ^2^ ^,^ ^3^. No Brasil, essa transição demográfica ocorre em meio a profundas desigualdades regionais e sociais ^4^. Entre 2010 e 2022, a população acima de 60 anos aumentou de 20,5 milhões para 32 milhões, passando de 10,8% para 15,1%, com projeção de alcançar 37,8% em 2070 ^5^.

Esse cenário de mudanças rápidas redefine estruturas sociais e econômicas, ampliando situações de vulnerabilidade social, familiar, comunitária e institucional ^6^ ^,^ ^7^. Entre essas manifestações, destaca-se a violência contra a pessoa idosa, definida pela Organização Mundial da Saúde como ato único ou repetido, bem como a omissão capaz de causar danos ou sofrimento a uma pessoa idosa em contextos de confiança. A violência contra a pessoa idosa abrange violência física, psicológica, sexual, financeira, negligência e abandono ^2^. Uma revisão sistemática estimou prevalência global anual de 15,7%, sobretudo de violência psicológica, financeira e por negligência ^8^. Por sua natureza multidimensional, compromete funcionalidade, qualidade de vida e aumenta a morbimortalidade, configurando importante problema de saúde pública ^8^ ^,^ ^9^ ^,^ ^10^.

A compreensão da violência contra pessoas idosas requer uma abordagem fundamentada nos determinantes sociais do envelhecimento ^11^. Baixa qualidade de vida, fragilidade funcional, dependência e isolamento social elevam a vulnerabilidade ao abuso ^12^. Um estudo multicêntrico brasileiro identificou associação inversa entre qualidade de vida e risco de violência, especialmente entre aqueles com pior percepção de bem-estar ^13^. O apoio social, efetivo ou percebido, também se destaca como fator protetivo ^11^ ^,^ ^14^. De modo semelhante, uma revisão sistemática aponta que baixos níveis de apoio social aumentam o risco de maus-tratos, sobretudo as formas de negligência e autonegligência ^15^. A fragilidade das relações sociais, o distanciamento intergeracional e a solidão intensificam esse risco ^16^.

Em contrapartida, fortalecer as interações sociais, seja pelo convívio social ou laboral, inclusive por meio da tecnologia, é uma via promissora. Estudos indicam que o uso de tecnologias digitais pode reduzir o isolamento e ampliar conexões e suporte social, e há evidências de que a participação social medeia parte do efeito do uso da internet sobre a saúde em pessoas idosas ^17^ ^,^ ^18^ ^,^ ^19^. No Brasil, o acesso digital entre pessoas idosas cresceu de 24,7%, em 2016, para 66%, em 2023, embora persistam marcantes desigualdades ^10^ ^,^ ^20^.

Apesar de avanços no campo de conhecimento, predominam estudos transversais e descritivos focados em dimensões isoladas da violência ^21^. Investigações longitudinais capazes de integrar fatores sociodemográficos, psicossociais, apoio social, interações sociais e qualidade de vida ainda são escassas ^12^ ^,^ ^21^. Essa lacuna limita a compreensão dos mecanismos que sustentam a violência e dificulta o direcionamento de políticas públicas para grupos mais vulneráveis ^22^ ^,^ ^23^ ^,^ ^24^.

Diante deste contexto, o objetivo desse estudo é estimar a associação entre o risco de violência e dimensões relacionadas às interações sociais, ao apoio social e à qualidade de vida entre os participantes do estudo longitudinal EpiFloripa Idoso.

## Método

### Desenho do estudo e população

Trata-se de um estudo com delineamento longitudinal prospectivo de base populacional. Utilizou-se dados da coorte EpiFloripa Idoso, que acompanha um segmento de pessoas idosas com 60 anos ou mais residentes na área urbana de Florianópolis, capital de Santa Catarina, Sul do Brasil. A onda 1 foi realizada em 2009/2010 (n = 1.702), a onda 2 em 2013/2014 (n = 1.197) e a onda 3 em 2017/2019 (n = 1.335).

A linha de base, conduzida em 2009/2010, utilizou amostragem por conglomerados em dois estágios, estratificada por setores censitários segundo décis de renda com seleção sistemática de domicílios no segundo estágio. O cálculo amostral com base em 44.460 pessoas idosas residentes em Florianópolis ^25^, uma prevalência para o desfecho desconhecida de 50%, erro de 4% e 95% de confiança. Aplicou-se efeito de delineamento (*deff* = 2), acréscimos de 20% para perdas e 15% para controle de confusão, estimando-se 1.599 participantes. Sendo sorteados sistematicamente cerca de 60 domicílios por setor censitário, incluindo-se todos os residentes com 60 anos ou mais. A amostra final alcançou 1.705 entrevistas (89,1% de resposta).

Na onda 2, foram elegíveis todas as pessoas idosas entrevistadas na linha de base que permaneciam vivas, identificados por investigação no Sistema de Informações sobre Mortalidade (SIM), totalizando 1.197 participantes e taxa de seguimento de 70,3% ^23^ ^,^ ^24^. A onda 3 foi composta por 956 participantes acompanhados desde a linha de base e que permaneceram elegíveis; indivíduos do estudo EpiFloripa Adulto que completaram 60 anos até julho de 2018; e novos participantes incluídos para reposição da amostra, todos residentes nos mesmos setores censitários originalmente sorteados, seguindo o mesmo plano amostral em múltiplos estágios. No total, 1.972 pessoas idosas foram elegíveis e 1.335 entrevistados (70% de resposta), com uma taxa de seguimento de 55,7% ^26^.

Detalhes metodológicos e operacionais encontram-se em publicações do estudo, apresentado por Schneider et al. ^23^ e Confortin et al. ^24^ ^,^ ^26^.

### Procedimentos

A coleta de dados foi realizada por meio de entrevistas face a face com o participante ou o seu informante nos três momentos: onda 1, onda 2 e onda 3. Os entrevistadores, treinados e habilitados, aplicaram um questionário com instrumentos validados de coleta de dados, composto por questões de múltiplas dimensões sociais e de saúde ^23^ ^,^ ^24^ ^,^ ^26^. Os questionários utilizados em cada onda estão disponíveis no site oficial da pesquisa: https://epifloripaidoso.paginas.ufsc.br/.

### Variável dependente

A variável dependente deste estudo foi o risco de violência avaliado em dois momentos, onda 2 (2013/2014) e onda 3 (2017/2019) a partir do uso do instrumento H-S/EAST (*Hwalek-Sengstock Elder Abuse Screening Test*) que rastreia situações e condições associadas à vulnerabilidade da pessoa idosa a essas formas de abuso, permitindo identificar risco de violência interpessoal de forma abrangente. O instrumento possui 15 itens em três dimensões: abuso direto (itens 4, 9, 10, 11 e 15), características de vulnerabilidade (itens 1, 3 e 6) e situação potencialmente abusiva (itens 2, 5, 7, 8, 12, 13 e 14), atribuindo-se um ponto para cada resposta afirmativa, com exceção dos itens 1, 6, 12 e 14, em que o ponto é aplicado para a resposta negativa. Para o presente estudo considerou-se risco de violência, três ou mais pontos na escala desse instrumento ^27^.

### Variáveis independentes

As variáveis independentes relacionadas à interação social foram o uso da internet, o trabalho remunerado e a participação em grupos sociais, todas dicotômicas (sim/não) e obtidas pelas perguntas: “Você usa internet ou e-mail?”, “O(a) Sr.(a) tem algum trabalho remunerado atualmente?” e “Você frequenta grupos de convivência ou religiosos?”.

Além disso, investigaram-se a autopercepção do apoio social e a qualidade de vida como exposições. O apoio social foi mensurado pela Escala MOS-SSS (*Social Support Scale of the Medical Outcomes Study*), composta por cinco dimensões (apoio material, afetivo, interação social positiva, emocional e informacional) e 27 itens com respostas em escala *Likert* de cinco pontos, gerando escores de 19 a 95, em que valores mais altos indicam maior apoio percebido. Para este estudo, adotou-se a mediana (≥ 73 pontos) como ponto de corte para classificar maior apoio social, dada a inexistência de valores de referência validados para populações específicas. Estratégia semelhante foi utilizada por Zhu et al. ^28^, que também empregaram a mediana para lidar com a heterogeneidade das distribuições do MOS-SSS.

A qualidade de vida foi avaliada pelo instrumento CASP-19 (*Control, Autonomy, Self-realization and Pleasure*), cuja estrutura métrica foi previamente examinada na onda 2 do EpiFloripa ^29^. As análises psicométricas indicaram duas dimensões: controle/autonomia (nove itens, escore 0-27, com seis itens reversos) e autorrealização/prazer (10 itens, escore 0-30). Escores mais elevados refletem melhor qualidade de vida. Para fins analíticos, ambas foram dicotomizadas pelo tercil superior (“sim”), adotando-se os seguintes pontos de corte: ≥ 25 pontos para controle/autonomia nas ondas 2 e 3; ≥ 28 (onda 2) e ≥ 29 pontos (onda 3) para autorrealização/prazer ^29^.

### Covariáveis

Variáveis sociodemográficas foram incluídas como covariáveis nos modelos estatísticos. As informações demográficas autorreferenciadas pelos participantes incluídas foram: sexo (masculino e feminino); faixa etária (60-69, 70-79, ≥ 80 anos); renda familiar *per capita* − salários mínimos brasileiros vigentes na data da entrevista (≤ 1, 1,1-5, 5,1-10 ou > 10) e escolaridade (sem escolaridade formal, 1-4, 5-8, 9-11, ≥ 12 anos). O salário mínimo variou de R$ 465,00 para R$ 510,00 em 2009/2010; de R$ 678,00 para R$ 724,00 em 2013/2014; e de R$ 937,00 para R$ 998,00 em 2017/2019, correspondendo aos valores oficiais vigentes nas datas das entrevistas.

### Análise estatística

Os dados descritivos foram apresentados por frequências absolutas e relativas, com intervalos de 95% de confiança (IC95%) calculados mediante uso dos pesos amostrais específicos de cada onda. Um modelo de análise longitudinal com equações de estimação generalizadas (GEE) foi utilizado para estimar os efeitos entre as variáveis independentes ou exposições (uso da internet, trabalho remunerado, participação em grupos sociais, apoio social e dimensões da qualidade de vida) e a variável dependente ou desfecho (risco de violência) ^30^.

Embora o delineamento seja longitudinal, o desfecho refere-se a uma condição dicotômica mensurada pelo H-S/EAST apenas nas ondas 2 e 3, e não a um evento incidente ao longo do seguimento. Dessa forma, não é possível estimar risco cumulativo ou razão de riscos, justificando a adoção de GEE com família binomial e função de ligação *logit*, que produzem *odds ratios* (OR) marginais apropriados para desfechos binários em medidas repetidas ^31^.

Os modelos GEE consideram a correlação intraindivíduo e produzem estimativas robustas em estudos longitudinais ^30^. Sendo assim, a onda 1 foi incluída como linha de base, permitindo utilizar todas as observações disponíveis mesmo com dados ausentes. O intervalo médio de aproximadamente quatro anos entre as ondas foi incorporado ao modelo para representar a estrutura temporal das observações. Os dados foram organizados no formato longo (*long format*) e estimaram-se dois modelos para cada variável independente: um modelo bruto (regressão bivariada) e um modelo ajustado por sexo, idade, renda familiar *per capita* e escolaridade.

A adequação dos modelos foi avaliada pela convergência e pela estabilidade das estimativas entre os modelos bruto e ajustado. Adotou-se nível de 5% de significância. As análises foram realizadas no software Stata 14.0 (https://www.stata.com).

## Aspectos éticos

A onda 1 do estudo EpiFloripa Idoso foi aprovada pelo Comitê de Ética em Pesquisa com Seres Humanos da Universidade Federal de Santa Catarina (parecer nº 352/08). A onda 3 (2017/2019) recebeu aprovação como uma emenda do EpiFloripa 2013/2014 (onda 2, CAAE: 16731313.0.0000.0121) ^26^. Todos os sujeitos concordaram em participar da pesquisa assinando o termo de consentimento livre e esclarecido e consentiram oralmente em receber a entrevistadora em seu domicílio.

## Resultados

A Tabela 1 apresenta as características da amostra nas ondas 1, 2 e 3 do estudo EpiFloripa Idoso. Observou-se predominância de mulheres em todas as coletas, variando de 62,5% na onda 1, 63,2% na onda 2 e 63% na onda 3. Ao longo do seguimento, houve aumento da proporção de participantes nas faixas etárias de 70 anos ou mais, reflexo do envelhecimento natural da coorte. Verificou-se também ampliação do nível de escolaridade, a proporção de participantes com ≥ 12 anos, cresceu de 25,2% na onda 1 para 33,5% na onda 3, a baixa escolaridade entre 0 e 4 anos de estudo manteve redução. Além de uma maior proporção de participantes com renda familiar *per capita* entre 1-5 salários mínimos, passando de 48,7% na onda 1, para 56% na onda 3, enquanto rendas ≤ 1 salário mínimo diminuíram no mesmo período (Tabela 1).

**Tabela 1:** Características sociodemográficas, econômicas e de condições de vida dos participantes do estudo EpiFloripa Idoso, segundo as ondas de coleta. Florianópolis, Santa Catarina, Brasil, 2009-2019.

Variável	Onda 1 (2009-2010)		Onda 2 (2013-2014)		Onda 3 (2017-2019)	
	n	% (IC95%)	n	% (IC95%)	n	% (IC95%)
Sexo	1.702		1.197		1.335	
Masculino	614	37,5 (34,8; 40,4)	419	38,8 (33,6; 40,2)	521	36,9 (33,8; 40,2)
Feminino	1.088	62,5 (59,5; 65,3)	778	63,2 (59,7; 66,4)	814	63,0 (59,8; 66,1)
Faixa etária (anos)	1.702		1.197		1.335	
60-69	848	50,7 (47,7; 53,5)	412	33,9 (30,2; 37,8)	461	30,2 (25,4; 35,6)
70-79	616	35,6 (32,7; 38,6)	509	42,8 (38,9; 46,8)	554	43,7 (39,1; 48,3)
≥ 80	238	13,7 (11,4; 16,5)	276	23,2 (20,0; 25,7)	320	26,0 (22,3; 30,2)
Escolaridade (anos de estudo)	1.695		1.194		1.330	
Sem escolaridade formal	159	8,2 (6,3; 10,5)	93	7,0 (5,4; 9,2)	62	4,4 (3,2; 6,1)
1-4	595	33,0 (28,5; 37,9)	430	33,9 (28,6; 39,0)	383	27,8 (22,6; 33,6)
5-8	307	17,6 (15,4; 20,4)	199	15,8 (13,6; 18,9)	226	18,0 (14,0; 22,8)
9-11	241	16,0 (13,6; 18,7)	180	17,2 (14,7; 20,1)	228	16,1 (13,4; 19,3)
≥ 12	393	25,2 (20,4; 30,2)	292	26,1 (21,2; 31,7)	431	33,5 (27,1; 40,5)
Renda familiar *per capita* (salários mínimos mensais)	1.702		1.147		1.334	
≤ 1	621	34,6 (30,5; 39,0)	309	24,2 (20,5; 28,4)	340	26,0 (22,5; 30,0)
1,1-5	840	48,7 (45,2; 52,1)	634	56,7 (51,9; 61,4)	767	56,0 (52,3; 59,6)
5,1-10	172	11,3 (8,9; 14,2)	137	10,9 (8,6; 13,7)	166	13,4 (10,4; 17,1)
> 10	69	5,4 (3,4; 8,4)	67	8,2 (5,0; 13,2)	61	4,5 (3,1; 6,5)
Risco de violência	-	-	1.137		1.249	
Não			765	68,6 (64,8; 72,1)	968	77,6 (74,8; 80,5)
Sim			372	31,4 (27,8; 35,2)	281	22,3 (19,1; 25,9)
Uso de internet	1.702		1.195		1.335	
Não	1.360	77,0 (72,3; 81,2)	877	71,0 (66,1; 75,3)	687	53,8 (48,0; 59,6)
Sim	342	23,0 (18,8; 27,6)	318	29,0 (24,7; 33,9)	648	46,1 (40,3;51,9)
Trabalho remunerado	1.702		1.090		1.248	
Não	1.474	86,6 (84,2; 88,7)	957	87,4 (84,1; 90,0)	1.093	88,0 (85,3; 90,3)
Sim	282	13,4 (11,2; 15,8)	133	12,6 (9,9; 15,9)	155	12,0 (9,7; 14,7)
Convivência em grupos	1.702		1.196		1.335	
Não	715	42,3 (38,9; 45,8)	709	60,3 (56,6; 63,8)	784	58,7 (54,7; 62,6)
Sim	987	57,7 (54,2; 61,0)	487	39,7 (36,1; 43,3)	551	41,2 (37,4; 45,2)
Apoio social	-	-	-	-	1.233	
Baixo					579	44,7 (40,3; 49,2)
Alto					654	55,2 (50,7; 59,6)
Qualidade de vida − controle/autonomia	-	-	1.134		1.261	
Baixa			804	71,2 (67,7; 74,5)	883	71,4 (67,1; 75,3)
Alta			380	28,8 (25,5; 32,3)	378	28,5 (24,6; 32,8)
Qualidade de vida − autorrealização/prazer	-	-	1.135		1.254	
Baixa			807	70,7 (67,4; 73,9)	897	71,1 (67,2; 74,7)
Alta			328	29,3 (26,2; 32,6)	357	28,9 (25,3; 32,7)

IC95%: intervalo de 95% de confiança estimados com peso amostrais (*svy*).

Em relação ao desfecho mensurado pelo H-S/EAST (ponto de corte ≥ 3), a maior parte dos participantes não foi classificada com risco de violência nas ondas do estudo. A prevalência do desfecho foi mais elevada na onda 2 (31,4%), reduzindo-se na onda 3 (22,3%). Observou-se ainda aumento expressivo do uso de internet ao longo da coorte (23% na onda 1; 29% na onda 2; 46,1% na onda 3), enquanto o trabalho remunerado e a participação em grupos sociais apresentaram redução entre as ondas 2 e 3 (Tabela 1).

A Tabela 2 apresenta as prevalências ponderadas do desfecho nas ondas 2 e 3 segundo características sociodemográficas, interação social, apoio social e qualidade de vida. Entre os sexos, observou-se maior prevalência de vulnerabilidade à violência no sexo feminino, com 32,6% (IC95%: 28,6; 36,9) na onda 2 e 24,5% (IC95%: 19,9; 29,7) na onda 3. A vulnerabilidade à violência aumentou progressivamente com idade, alcançando a maior prevalência entre pessoas com ≥ 80 anos na onda 2 (35,4%; IC95%: 27,9; 43,7). Embora tenha diminuído na onda 3, o percentual permaneceu elevado nesse grupo etário (22,1%; IC95%: 15,9; 29,9).

**Tabela 2:** Prevalência do risco de violência segundo características sociodemográficas, participação em grupos sociais, trabalho remunerado, uso de internet, apoio social e qualidade de vida do estudo EpiFloripa Idoso (ondas 2 e 3). Florianópolis, Santa Catarina, Brasil, 2009-2019.

Variável	Onda 2		Onda 3	
	n	% (IC95%)	n	% (IC95%)
Sexo	372		281	
Masculino	125	29,4 (24,4; 34,8)	102	18,7 (14,4; 23,8)
Feminino	247	32,6 (28,6; 36,9)	179	24,5 (19,9; 29,7)
Faixa etária (anos)	372		281	
60-69	117	27,5 (23,1; 32,4)	119	29,4 (22,5; 37,2)
70-79	164	32,6 (27,0; 38,7)	106	17,4 (13,0; 23,0)
≥ 80	91	35,4 (27,9; 43,7)	56	22,1 (15,9; 29,9)
Escolaridade (anos de estudo)	371		279	
Sem escolaridade formal	39	42,6 (29,7; 56,6)	12	18,0 (8,2; 34,9)
1-4	138	32,4 (27,0; 38,3)	72	19,9 (15,0; 25,9)
5-8	58	30,9 (23,6; 39,3)	51	28,7 (19,4; 40,3)
9-11	49	24,0 (16,6; 33,4)	54	24,6 (16,9; 34,3)
≥ 12	87	32,4 (25,6; 40,0)	90	20,1(15,9; 25,1)
Renda familiar *per capita* (salários mínimos mensais)	357		281	
≤ 1	113	36,1 (29,8; 43,0)	80	22,8 (16,8; 30,2)
1,1-5	187	29,2 (25,0; 33,7)	162	24,7 (20,0; 30,1)
5,1-10	36	28,4 (20,5; 38,0)	29	13,8 (8,3; 22,1)
> 10	21	35,2 (20,3; 53,6)	10	14,1 (6,0; 29,8)
Participação em grupos sociais	372		281	
Não	225	34,6 (30,2; 39,3)	182	25,2 (21,2; 29,8)
Sim	147	26,7 (21,5; 32,5)	99	18,4 (14,2; 23,4)
Trabalho remunerado	338		260	
Não	313	33,1 (28,7; 38,0)	227	21,5 (18,3; 25,1)
Sim	25	20,4 (13,4;29,8)	33	28,3 (17,5; 42,3)
Uso de internet	372		281	
Não	286	34,5 (30,2; 39,0)	136	23,1 (18,3; 28,7)
Sim	86	24,3 (20,1; 29,2)	145	21,5 (17,1; 26,5)
Apoio social *	-		267	
Não	-	-	167	26,6 (21,8; 32,0)
Sim	-	-	100	18,3 (13,3; 24,7)
Qualidade de vida − controle/autonomia **	368		276	
Não	327	39,0 (34,9; 43,3)	230	26,9 (22,9; 31,3)
Sim	41	11,3 (8,3; 15,5)	46	10,8 (7,7; 15,0)
Qualidade de vida − autorrealização/prazer **	370		276	
Não	305	36,9 (32,4; 41,6)	232	26,7 (22,8; 31,0)
Sim	65	17,8 (13,2; 23,3)	44	12,4 (7,7; 19,4)

IC95%: intervalo de 95% de confiança; n: número de entrevistados (não ponderado).

Nota: prevalência e IC95% estimados com pesos amostrais (*svy*);

* Foi utilizado como ponto de corte a mediana, onde valores ≥ mediana foram classificados como “sim” (apoio social percebido) e valores < mediana foram classificados como “não” (sem percepção de apoio social);

** Qualidade de vida: (sim) − tercil superior − indicam níveis maiores de qualidade de vida e (não) tercil inferior - menores níveis de qualidade de vida.

Quanto à escolaridade, participantes sem instrução formal apresentaram as prevalências mais elevadas na onda 2, com 42,6% (IC95%: 29,7; 56,6). Na onda 3, entretanto, observou-se um padrão distinto, com maior prevalência entre pessoas com 5 a 8 anos de estudo (28,7%). Relativo ao aspecto financeiro, no estrato de renda mais baixa (≤ 1 salário mínimo), observou-se a maior prevalência de risco de violência na onda 2, com 36,1% (IC95%: 29,8; 43,0), seguida de redução para 22,8% (IC95%: 16,8; 30,2) na onda 3 (Tabela 2).

Entre as variáveis de interação social, a participação em grupos esteve consistentemente associada a menores prevalências do desfecho (26,7% na onda 2 e 18,4% na onda 3). Para os participantes que exerciam o trabalho, o desfecho foi menor na onda 2, atingindo 20,4% (IC95%: 13,4; 29,8), contudo, na onda 3, houve um aumento de vulnerabilidade à violência para 28,3% (IC95%: 17,5; 42,3). Quanto ao uso de internet, registrou-se menor prevalência entre usuários na onda 2 (24,3% contra 34,5% entre não usuários), porém, na onda 3, as prevalências foram semelhantes entre os grupos (Tabela 2).

O apoio social, avaliado na onda 3, demonstrou que os participantes categorizados sem percepção de apoio social tiveram maior prevalência do desfecho (26,6%) em comparação àqueles com maior percepção de apoio (18,3%). Nas dimensões da qualidade de vida, verificou-se que, na dimensão controle/autonomia, participantes com menores escores apresentaram prevalências de vulnerabilidade à violência de 39,0%, na onda 2, e 26,9%, na onda 3, enquanto aqueles com escores mais elevados mostraram prevalências menores, de 11,3% e 10,8%, respectivamente. De forma semelhante, na dimensão autorrealização/prazer, as prevalências foram de 36,9% e 26,7% entre indivíduos com escores baixos, em contraste com 17,8% e 12,4% entre os participantes com melhores níveis nessas dimensões (Tabela 2).

Na Tabela 3, a análise multivariada demonstrou associações consistentes entre as variáveis de interação social, apoio social e qualidade de vida com a ocorrência do desfecho. O trabalho remunerado mostrou-se um fator protetor, reduzindo a vulnerabilidade à violência (OR = 0,68; IC95%: 0,49; 0,93), assim como a participação em grupos sociais (OR = 0,75; IC95%: 0,62; 0,92). O uso de internet apresentou direção semelhante entre as análises, embora sem significância estatística no modelo ajustado (OR = 0,80; IC95%: 0,63; 1,02) (Tabela 3).

**Tabela 3:** Associação entre uso de internet, trabalho remunerado, participação em grupos sociais, apoio social, qualidade de vida com o risco de violência em pessoas idosas do estudo EpiFloripa Idoso. Florianópolis, Santa Catarina, Brasil, 2009-2019.

Variável exposição	Análise bruta			Análise ajustada *		
	OR	IC95%	Valor de p	OR	IC95%	Valor de p
Uso de internet	0,78	0,64; 0,95	0,014	0,80	0,63; 1,02	0,070
Trabalho remunerado	0,67	0,49; 0,91	0,012	0,68	0,49; 0,93	< 0,019
Participação em grupos	0,76	0,63; 0,92	< 0,005	0,75	0,62; 0,92	< 0,005
Apoio social	0,43	0,33; 0,58	< 0,001	0,44	0,33; 0,59	< 0,001
Qualidade de vida − controle/Autonomia	0,29	0,22; 0,37	< 0,001	0,30	0,23; 0,38	< 0,001
Qualidade de vida − autorrealização/prazer	0,43	0,34; 0,54	< 0,001	0,44	0,34; 0,55	< 0,001

OR: *odds ratio*; IC95%: intervalo de 95% de confiança.

Nota: para todas as variáveis dicotômicas, adotou-se como referência a categoria “Não”, por representar a ausência da condição (não usar internet, não ter trabalho remunerado, não participar de grupos sociais, ausência de apoio social e menor qualidade de vida).

* Modelos ajustados por sexo, idade, renda familiar *per capit*a e escolaridade.

O apoio social mostrou uma associação forte, com menor ocorrência de violência entre participantes com maior percepção de apoio (OR = 0,44; IC95%: 0,33; 0,59). As dimensões de qualidade de vida apresentaram as magnitudes mais expressivas: indivíduos com maiores níveis de controle/autonomia tiveram OR = 0,30 (IC95%: 0,23; 0,38), enquanto aqueles com escores mais elevados em autorrealização/prazer apresentaram OR = 0,44 (IC95%: 0,34; 0,55). Esses resultados indicam que os fatores estimados se associaram de forma consistente à menor ocorrência do desfecho ao longo do seguimento (Tabela 3).

## Discussão

Neste estudo, identificou-se que a prevalência de risco de violência, segundo o H-S/EAST, reduziu da onda 2 (31,4%) para a onda 3 (22,3%), embora os valores tenham permanecido elevados. Esses valores superam a estimativa global de 15,7% apresentada em uma revisão sistemática com metanálise ^32^. No contexto brasileiro, estudos apontam prevalências entre 1,6% a 20,2%, predominando formas não físicas, como violência psicológica, verbal e negligência, tipos mais frequentes e de difícil notificação ^19^ ^,^ ^33^. A redução observada ao longo do seguimento pode refletir mudanças no acesso a recursos comunitários, na proteção social ou no próprio curso do envelhecimento ^33^ ^,^ ^34^.

Os achados deste estudo também demonstram as desigualdades sociais que ampliam a vulnerabilidade à violência, com maiores prevalências entre mulheres, indivíduos com menor escolaridade e pessoas nas faixas mais baixas de renda *per capita*, um perfil de desigualdade evidenciado na literatura nacional e internacional ^35^.

As interações sociais mostraram associações relevantes com o desfecho. Entre essas interações destaca-se a inclusão digital. Na análise descritiva, observou-se menor prevalência (24,3%) entre usuários que utilizaram internet na onda 2 em comparação aos que informaram não utilizar, embora essa diferença não se manteve na onda 3. Na análise multivariada, o uso da internet perdeu significância após ajuste, contudo, a estabilidade do efeito (OR = 0,80) demonstrou atuar como um fator de proteção, esse efeito demonstra limitação, especialmente para as pessoas idosas, que muitas vezes enfrentam barreiras relacionadas ao acesso às tecnologias e à alfabetização digital ^36^ ^,^ ^37^.

A participação em grupos sociais destacou-se pela estabilidade da associação (OR = 0,75), indicando papel protetivo do engajamento social ^38^ ^,^ ^39^. O trabalho remunerado apresentou efeito protetor na onda 2, atenuado na onda 3, mas preservado nos modelos longitudinais (OR ajustado = 0,68; IC95%: 0,49; 0,93), resultado compatível com estudos que relacionam inserção produtiva à autonomia, autoestima e maior integração social ^28^ ^,^ ^38^ ^,^ ^39^. A continuidade laboral no envelhecimento pode fortalecer realização pessoal, vida ativa e inclusão social, com menor foco financeiro, frequentemente associando-se a atividades informais e ao aumento da percepção de apoio social ^38^. A inserção social promovida pelo trabalho conecta-se diretamente à percepção de apoio social ^39^.

O apoio social neste estudo apresentou associação protetora (OR = 0,44), achado consistente com um estudo internacional multicêntrico realizado em instituições de longa permanência ^40^ e com uma revisão abrangendo países da América do Norte, Europa ^41^ e Ásia ^42^ que demonstram que redes sociais fortalecidas atuam como barreiras contra maus-tratos ^40^ ^,^ ^41^ ^,^ ^42^. 

As dimensões de qualidade de vida apresentaram forte associação com a vulnerabilidade à violência. Pessoas idosas com menores escores de controle/autonomia (OR = 0,30) e de autorrealização/prazer (OR = 0,44) exibiram prevalências mais elevadas do desfecho, e essas relações mantiveram magnitude expressiva após ajuste. Evidências internacionais corroboram que baixa qualidade de vida e maior dependência física e psicológica ampliam a vulnerabilidade à violência ^43^ ^,^ ^44^. Além disso, determinantes sociais da saúde como, renda, escolaridade, acesso a serviços e participação social influenciam diretamente o bem-estar e a segurança, constituindo bases essenciais para um envelhecimento protegido ^45^ ^,^ ^46^. Esses achados reforçam o papel de fatores biopsicossociais na redução da exposição à violência em pessoas idosas ^47^ ^,^ ^48^ ^,^ ^49^.

É possível referir algumas limitações neste estudo, a natureza subjetiva dos dados autorreferidos que pode influenciar a precisão das informações relatadas, além do viés de seleção, uma vez que a amostra não contempla pessoas idosas hospitalizadas ou residentes em instituições de longa permanência ^50^. Contudo, tais estratégias são amplamente aceitas em pesquisas longitudinais e não comprometem a validade interna das estimativas ^21^ ^,^ ^51^.

A utilização dos dados da coorte EpiFloripa Idoso, reconhecida pela robustez metodológica e representatividade populacional, fortalece a validade dos achados. Os fatores de proteção identificados fornecem subsídios para orientar estratégias intersetoriais que atuem não apenas na resposta à violência já estabelecida, mas também na mitigação das vulnerabilidades que a precedem.

## Conclusão

Este estudo demonstrou que maiores níveis de interações sociais, de apoio social percebido e qualidade de vida atuam como fatores protetores contra situações de maus-tratos e abusos em pessoas idosas. Os modelos longitudinais GEE mostraram, de forma consistente, que esses determinantes sociais e subjetivos mantêm efeitos protetores ao longo do tempo, reduzindo a vulnerabilidade à violência. 

A principal contribuição deste estudo reside na abordagem inovadora que integra múltiplos fatores sociais, relacionais e de bem-estar em um modelo longitudinal de base populacional envolvendo o tema da violência contra pessoas idosas, abordagem ainda pouco estudada no contexto brasileiro. Contudo, permanece a necessidade de aprofundar investigações sobre esses determinantes, sobretudo em cenários de maior vulnerabilidade e menor disponibilidade de dados epidemiológicos.

## Data Availability

As fontes de informação utilizadas no estudo estão indicadas no corpo do artigo.
